# Protective Effect of High Adherence to Mediterranean Diet on the Risk of Incident Type-2 Diabetes in Subjects with MAFLD: The Di@bet.es Study

**DOI:** 10.3390/nu16213788

**Published:** 2024-11-04

**Authors:** Ana Lago-Sampedro, Wasima Oualla-Bachiri, Sara García-Serrano, Cristina Maldonado-Araque, Sergio Valdés, Viyey Doulatram-Gamgaram, Gabriel Olveira, Elias Delgado, Felipe Javier Chaves, Luis Castaño, Alfonso Calle-Pascual, Josep Franch-Nadal, Gemma Rojo-Martínez, Eva García-Escobar

**Affiliations:** 1 Centro de Investigaciónn Biomedica en Red de Diabetes y Enfermedades Metabolicas Asociadas (CIBERDEM), Instituto de Salud Carlos III, 28029 Madrid, Spainfelipe.chaves@uv.es (F.J.C.); acallepascual@hotmail.com (A.C.-P.); josep.franch@gmail.com (J.F.-N.); 2 UGC Endocrinología y Nutrición, Hospital Regional Universitario de Málaga, IBIMA Plataforma BIONAND, 29009 Málaga, Spain; 3 Departamento de Medicina y Dermatología, Universidad de Málaga-UMA, 29071 Málaga, Spain; 4 Centro de Investigaciónn Biomedica en Red de Enfermedades Raras (CIBERER), Instituto de Salud Carlos III, 28029 Madrid, Spain; eliasdelga@gmail.com; 5 Department of Endocrinology and Nutrition, Health Research Institute of the Principality of Asturias (ISPA), Central University Hospital of Asturias, University of Oviedo, 33011 Oviedo, Spain; 6 Genomic and Genetic Diagnosis Unit, INCLIVA Biomedical Research Institute, 46010 Valencia, Spain; 7 Biobizkaia Health Research Institute, Hospital Universitario Cruces, University of the Basque Country, CIBERDEM, CIBERER, Endo-ERN, 48903 Barakaldo, Spain; 8 Department of Endocrinology and Nutrition, San Carlos University Hospital of Madrid, 28040 Madrid, Spain; 9 EAP Raval Sud, Catalan Institute of Health, GEDAPS Network, Primary Care, Research Support Unit (IDIAP—Jordi Gol Foundation), 08001 Barcelona, Spain

**Keywords:** Mediterranean diet, protecting factor, Type-2 diabetes incidence, MAFLD

## Abstract

**Background/Objectives**: Metabolic Dysfunction-Associated Fatty Liver Disease (MAFLD) increases the risk of Type-2 Diabetes (T2DM). The Mediterranean diet (MD) has shown advantages in the management of MAFLD and preventing co-morbidities; however, its relationship with T2DM development in MAFLD has been less investigated. We aimed to evaluate the association of MD adherence with the risk of incident T2DM in the Spanish adult population with MAFLD and according to their weight gain at 7.5 years follow-up. **Methods**: A cohort of 714 participants (without weight increment: 377; with weight increment: 337) from the Di@bet.es cohort study with MAFLD and without T2DM at baseline were investigated. Anthropometric, sociodemographic, clinical data, and a survey on habits were recorded. OGTT and fasting blood biochemistry determinations were made. Baseline adherence to MD was estimated by the adapted 14-point MEDAS questionnaire and categorized as high and low adherence. **Results**: In total, 98 people developed T2DM at follow-up. The high adherence to MD was inversely associated with the development of T2DM in both the overall population (0.52 [0.31–0.87]) and subjects without weight gain at follow-up (0.35 [0.16–0.78]). **Conclusions**: Our results suggest the protective effect of high adherence to MD regarding the risk of T2DM in subjects with MAFLD, with this health benefit being more evident in men with the absence of weight gain. These results support the recommendations for MD use in these patients.

## 1. Introduction

Non-alcoholic fatty liver disease (NAFLD) is a highly prevalent liver disease with an overall worldwide prevalence in the adult population of about 25%, a value that is substantially increased in individuals with type 2 diabetes (T2DM) [1]. Specifically in Spain, an NAFLD prevalence of 26% among the adult population has been estimated [2]. Due to the dysmetabolic co-morbidities that commonly affect NAFLD patients, new nomenclature and definition have been proposed for NAFLD [3,4]. The new term, Metabolic Dysfunction-Associated Fatty Liver Disease (MAFLD), encompasses subjects with evidence of liver steatosis and at least one of the following three conditions: overweight/obesity; T2DM; or metabolic dysfunction [3,4]. MAFLD is more prevalent than NAFLD, with two-fifths of individuals meeting the diagnosis criteria [5], and it also presents different clinical characteristics with significantly higher rates of T2DM, hypertriglyceridemia, hypertension, and fibrosis risk among MAFLD than NAFLD patients [5]. MAFLD not only leads to increased liver-related morbidity and mortality but also to increased overall mortality and is associated with a greater risk of cardiovascular death [6].

Growing epidemiological data suggest a reciprocal relationship between MAFLD and T2DM [7,8]. T2DM is considered an important risk factor for MAFLD independently of obesity [9], and in addition, individuals with diagnosed MAFLD have a more than twofold increased risk of T2DM [10,11,12,13]. Given the intricate inter-relationship and the high global rates of MAFLD and T2DM, regular screening programs and prevention strategies for T2DM in patients with liver steatosis have been suggested as being useful for reducing its associated mortality and morbidity [8,14].

The most effective treatment for fatty liver to date is weight loss, so lifestyle interventions aimed at this weight reduction, including changes in diet and/or physical activity, are the primary option for the treatment and prevention of MAFDL [15,16]. Among different dietary patterns, the effects of the Mediterranean diet (MD) on MAFLD have been the most widely researched [9,15,16]. MD is characterized by a high intake of fruits, vegetables, olive oil, cereals, legumes, and fish, while moderate amounts of dairy, eggs, red wine, and a limited amount of red meat are consumed [17]. Observational and interventional studies have demonstrated that MD is beneficial for the management of MAFLD; the antioxidant, anti-inflammatory, and antimicrobial effects of MD have been suggested as a potential underlying mechanism for the improvement in metabolic outcomes [9,16]. In this context, several professional societies have recommended MD as the dietary pattern of choice to ameliorate liver stiffness and prevent the progression to advanced hepatic diseases [18]. Adherence to MD has an established benefit in long-term weight reduction regarding a low-fat diet [19], while low adherence to MD has been associated with the degree of hepatic fibrosis assessed by transient electrography in a study aimed to assess the relationship between adherence to the MD and inflammatory biomarkers in 40 patients with MAFLD [20]. Even without weight reduction, MD is able to improve metabolic status and steatosis, as shown in different studies [21,22,23].

Several authors have reported that fatty liver management with MD not only ameliorated hepatic steatosis and inflammation but also showed advantage in the prevention of co-morbidities such as insulin resistance, visceral obesity, dyslipidemia, and chronic inflammation [9,24]; however, despite the association between the presence of MAFLD and the risk of incident T2DM and the importance for the prevention of the co-morbidity development associated to fatty liver, to our knowledge, the relationship between MD adherence and T2DM onset in subjects with MAFLD has not been evaluated so far, and limited studies have investigated it in subjects with NAFLD [25]. Therefore, the aim of this study was to evaluate the potentially protective effect of the Mediterranean diet on the risk of incident T2DM at 7.5 years in the Spanish adult population with MAFLD and its relationship with weight increase.

## 2. Materials and Methods

### 2.1. Study Design, Setting, and Population

This is a secondary analysis from the population-based cohort study Di@bet.es epidemiological trial based on a subsample from the original cohort.

**Figure 1:**
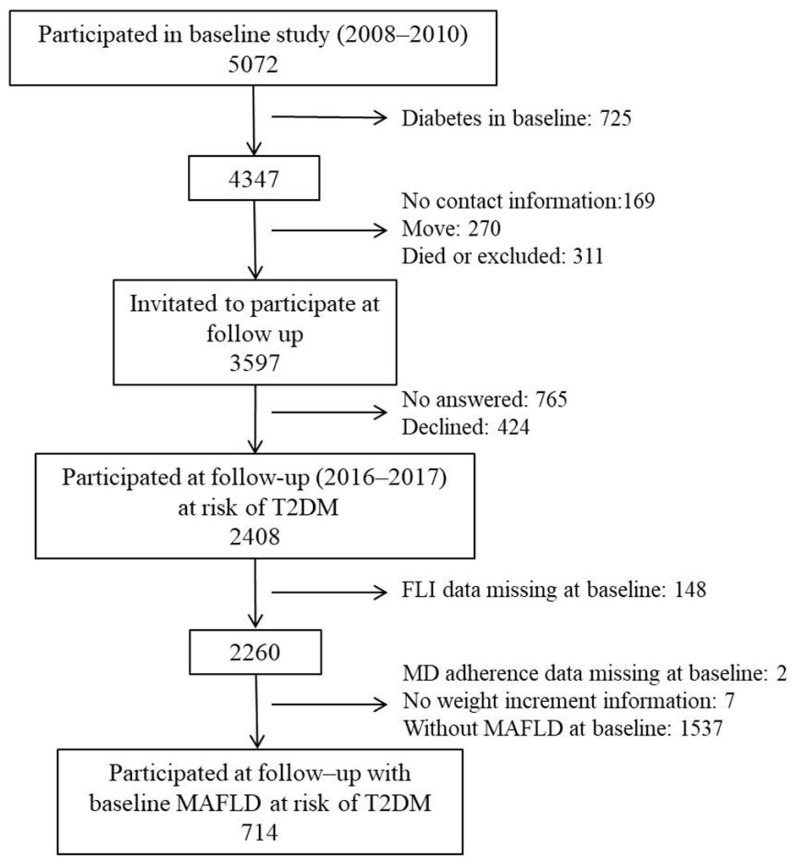
Flow diagram of the cohort study.

The initial cross-sectional study of Di@bet.es was conducted between 2008 and 2010 in a random cluster sampling of the Spanish population [26]. Detailed information on the participant flow chart and methodology of the Di@bet.es cohort has been previously published [27]. In short, the Di@bet.es study sample consisted of 5072 adults randomly selected from National Health System registries distributed into 100 clusters. The cohort was re-evaluated in 2016–2017 (7.5 ± 0.6 years follow-up). All subjects who had completed the baseline study were invited by letter and phone to attend another clinical examination, from which 725 had T2DM in a cross-sectional study. As with the cross-sectional study, people with serious illness, pregnancy, recent delivery or lactation, or surgery within the previous month were excluded, and finally, 2408 subjects without T2DM at baseline completed the follow-up. Baseline MAFLD was evaluated within these subjects. For the present sub-study, only followed-up participants at risk of T2DM with MAFLD, from which adherence to the MD was possible to determine, were included in the analyses (n = 714) (Figure 1).

MAFLD diagnosis was based on the coexistence of hepatic steatosis and one of the following criteria: overweight/obesity or metabolic dysfunctions. Liver steatosis was estimated by the use of the fatty liver index (FLI) [28]. The fatty liver index, a simple and accurate predictor of hepatic steatosis in the general population [28], a non-invasive and well-established method for the diagnosis of fatty liver against ultrasound [29,30] in both Asian and Western populations, has also been validated as an MAFLD marker against abdominal computed tomography [31]. An FLI value over 60 is established to confirm the presence of liver steatosis [28]. The presence of metabolic abnormalities was defined as the presence of at least two of the following: abdominal obesity (waist circumference ≥90 cm or ≥80 cm for male and females respectively); hypertension (blood pressure ≥ 130/85 mmHg or specific drug treatment); dyslipidemia (serum triglyceride ≥ 150 mg/dL or HDL-cholesterol <40 mg/dL for males and <50 mg/L for females or specific drug treatment); prediabetes (fasting plasma glucose ≥ 100 mg/dL and/or post OGTT glucose level ≥140 and <200 mg/dL); insulin resistance (HOMA levels over the 75th percentile of our population excluding subjects with T2DM); or serum C-reactive protein (CRP) > 2 mg/L [3].

For each subject, changes in weight (continuous variable), from baseline to follow-up study, were evaluated as the difference in weight values measured in both study stages (weight at follow-up visit—weight at baseline visit). The study population was also classified according to their weight gain at follow-up as without weight increment (changes in weight equal to or less than 0; n = 377) and with weight increment (changes in weight over 0; n =337).

This research was carried out in accordance with the Declaration of Helsinki (WHO 2011) of the World Medical Association. All participants provided written informed consent. This study was approved by the Ethics and Clinical Investigation Committee of the Hospital Regional Universitario de Málaga, Spain Codes CEI20070612, date 12 June 2007; CEI20110324, date 24 March 2011; CEI20161026 date 26 October 2016; CEI21022019 date 21 February 2019) and the Comisión Central de Investigación de Atención Primaria (acta 03/17), in addition to other regional ethics and clinical investigation committees all over Spain.

### 2.2. Variables and Procedures

The participants were invited to a single examination visit at their health facility during both phases of this study (one visit at baseline and a second visit at follow-up). Participants were attended by a nurse who had been appropriately trained for this project. A structured questionnaire provided by the interviewer was used to gather information. A physical examination, blood sample collection, and an oral glucose tolerance test (OGTT) were also performed.

Clinical variables such as blood pressure levels, fasting serum levels of glucose, insulin, blood lipids, and transaminase levels, among other clinical variables, were determined by routine methods.

The anthropometric, sociodemographic, and lifestyle factors such as age (ranged 18–30, 31–45, 46–60, 61–75, >75), sex, weight, height, waist and hip circumferences, family history of T2DM (yes/no), alcohol consumption (never: no alcohol consumption, low: <1 serving/week, moderate: 1–2 servings/day for men and 1 serving/day for women; and high: >2 servings/day for men and over 1 serving/day for women), physical activity (according to the IPAQ questionnaire [32] as low, moderate, and high), or smoking habits (current smokers vs. former/never been smokers) were recorded. The use of steatogenic medications [33] (amiodarone, methotrexate, tamoxifen, fluoxetine, valproic acid, acetylsalicylic acid, or non-steroidal anti-inflammatory drugs) was also considered.

The adapted 14-point MedDiet adherence screener (MEDAS) questionnaire [34] was used to estimate adherence to the MD after applying a qualitative food frequency questionnaire of 50 food items. The cut-off point of 9 was established to define high or low MD adherence categories [35].

BMI was calculated as weight/height^2^. A BMI equal to or higher than 30 kg/m^2^ was used to define the presence of obesity. Insulin resistance was estimated by the homeostasis model assessment (HOMA), and the HOMA 75th percentile of the total population excluding subjects with T2DM was calculated as the cut-off for insulin resistance risk category (HOMA-IR as yes/no) [36].

### 2.3. Definition of New Cases of T2DM

New cases of T2DM at follow-up were diagnosed according to the presence of at least one of the following at follow-up:

Fasting serum glucose equal to or higher than 126 mg/dL; 2 h post-OGTT equal to or higher than 200 mg/dL; HbA1c equal to or higher than 6.5%; or use of glucose-lowering medication at the follow-up examination [27].

### 2.4. Statistical Analysis

Data in tables are presented as mean ± SD, proportions or Odd ratios, and their corresponding 95% confidence intervals (OR (95%CI)). Normal distribution was tested through a P-P plot, and equality of variances was tested through Levene’s test. Log-transformation of variables not following a normal distribution was performed in order to include them in the ANOVA analyses. Differences in baseline categorical or continuous variables according to the MD adherence were tested with a chi-squared test or ANOVA (adjusted by sex, age, and abdominal obesity), respectively. OR (95%CI) for the association between MD adherence and T2DM development was evaluated through logistic regression analysis adjusted by potential confounders such as age, sex, abdominal obesity, weight gain, fasting glucose levels, family history of T2DM, insulin resistance, hypertension or dyslipidemia, besides lifestyle variables (smoking habits, alcohol consumption or physical activity) and steatogenic medication. MD analysis of the condition of intermediate or collider as appropriate for covariates included in the logistic regression models was performed to avoid possible statistical bias or false estimates during the analysis. The goodness of fit of the logistic regression models was assessed through the Hosmer–Lemeshow test. *p* < 0.05 (two-sided) was considered statistically significant. Interaction analysis between MD adherence and weight gain was evaluated in the multiplicative and additive scales. The multiplicative interaction effect was studied by including the product term in the fully adjusted regression models, while the additive interaction effect was assessed by the estimation of the relative excess risk due to interaction (RERI), attributable proportion (AP), and the synergy index (SI) as standard indices to detect the presence of additive interaction [37]. All statistical analyses were performed with R software (v. 2023.12.1).

## 3. Results

### 3.1. Baseline Characteristics of the Population

The baseline general characteristics of the overall population according to adherence to MD categories and their weight gain at follow-up are presented in Table 1. The study sample included 714 individuals with a mean age of 52 years (age range 18–89 years), from which 55.7% were men. As was expected for the inclusion criteria, a high percentage of our general population presented metabolic disturbances such as metabolic syndrome, insulin resistance, dyslipidemia, abdominal obesity, or hypertension.

The total sample mean of weight change was −0.81 kg (range −38.0 to 43.6 kg), with 52.8% of the individuals (n = 377) reducing or maintaining their weight at follow-up.

Adherence to the Mediterranean diet, adjusted by sex, age, and abdominal obesity as appropriate, was inversely associated with age, weight, BMI, waist circumference, presence of insulin resistance, and physical activity in the overall population and the group of subjects without weight increment at follow-up (Table 1). Compared to the low adherence category, subjects with high adherence to MD were older (*p* < 0.001), with significantly lower values of BMI (*p* < 0.01), weight (*p* < 0.01), and waist circumference (*p* < 0.01). Although no differences in HOMA index levels were found between adherence to MD categories, the percentage of individuals with insulin resistance among the group of subjects with high adherence to MD was significantly lower than those with low adherence to MD (*p* = 0.02). Regarding physical activity, the individuals with low adherence to MD were more sedentary (SF-IPAQ category = Low) than those with high adherence to MD, and the percentage of individuals who practiced moderate levels of physical activity was higher in the high adherence to MD group (*p* = 0.04).

Baseline general clinical characteristics of the population stratified by weight gain and according to the adherence to MD are presented in Table 2. Within the group of subjects without weight increment at follow-up, those individuals with high adherence to MD showed higher baseline levels of the hepatic enzymes GOT (*p* = 0.01) and GGT (*p* = 0.02) compared to those with low adherence. Alternatively, the percentage of subjects with prediabetes was directly associated with adherence to MD in the group of subjects with weight increment at follow-up (*p* = 0.02).

### 3.2. New Onset of T2DM

The percentages of subjects who develop T2DM according to the adherence to MD in the complete population and stratified by sex and weight gain at follow-up are presented in Table 3.

In our population with MAFLD, the number of individuals who developed T2DM after 7.5 years of follow-up was 98 people; among them, 70 reported low adherence to the MD at baseline. In all cases, the complete population and stratified by sex or weight increment, the percentage of incident T2DM was higher in those subjects with lower adherence to MD; nevertheless, only in the group of subjects without weight increment at follow-up, the differences in the proportions of new cases of T2DM according to adherence to MD reach significance (*p* = 0.02; Table 3).

### 3.3. Adherence to Mediterranean Diet as a Protecting Factor for T2DM Development

Multivariable logistic regression analyses for the development of T2DM at 7.5 years of follow-up according to the level of adherence to MD at baseline were performed.

**Figure 2:**
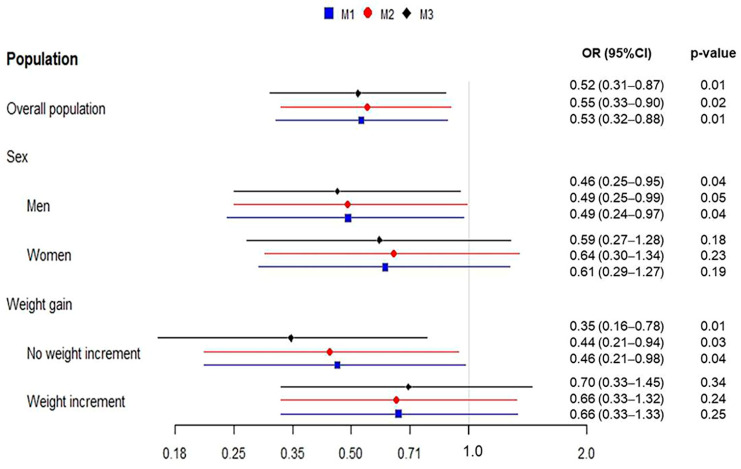
Odd ratios forest plot of association between adherence to Mediterranean diet (high vs. low) and incident T2DM in overall population and stratified by sex and weight gain at follow-up. Dots and bars are ORs and 95% CI for incident T2DM derived from multiple logistic regression analyses. M1: Logistic regression model for the risk of T2DM incidence adjusted by sex (except in the sex-based analysis), age, abdominal obesity, weight gain (except in the weight gain-based analysis), fasting serum glucose levels, and family history of T2DM. M2: M1 + insulin resistance index, hypertension, dyslipidemia, and steatogenic medication. M3: M2 + lifestyle variables (smoking habits, alcohol consumption, and physical activity).

In the overall population, results from these adjusted multivariable models showed that the high adherence to MD was, in all cases, a significant independent protecting factor for the development of T2DM, with almost two times less likelihood of new onset of T2DM among subjects with high adherence to MD compared to those with low adherence (Figure 2). Sex-based stratification analysis showed a protector effect of high adherence to MD in both, although the significant inverse association was only retained in men (Figure 2). The use of weight gain as a continuous variable instead of dichotomic did not modify these associations (Supplementary Table S1).

Additionally, multivariable logistic regression analyses were repeated in the population stratified by weight gain at follow-up categories (Figure 2). According to these models, adherence to MD appeared significant and inversely associated with new cases of T2DM only in the group of subjects without weight gain at follow-up, which showed 2.17 to 2.9 times less likelihood of developing T2DM compared to those with low adherence (Figure 2). To more deeply investigate the possible interaction effect of weight gain on the relationship between MD adherence and incident T2DM, multiplicative and additive interactions were evaluated in the fully adjusted model. No significant association between multiplicative interaction term and incident T2DM was found (1.72 [0.62–4.74], *p* = 0.29). Also, no significant association between the additive interaction and the new onset of T2DM was found, with RERI, AP, and SI values of −0.65 (−4.53, 1.49), −0.24 (−1.34, 0.35), and 0.73 (0.3, 1.78), respectively. The inclusion of the interaction terms in the logistic regression models did not alter the significant association between MD adherence and incident T2DM.

## 4. Discussion

The findings presented herein reveal an independent protective role of MD against T2DM development in a large cohort of the Spanish adult population with MAFLD, with a more apparent effect in men and those individuals without weight gain at 7.5 years of follow-up. This inverse association was independent of different T2DM risk factors such as age, sex, adiposity, fasting glucose levels, family history of T2DM, HOMA-IR, plasma lipids, hypertension, or other lifestyle variables.

A number of studies have suggested a two-way relationship between MAFLD and T2DM, probably linked to the insulin resistance condition [7,8]. Previous studies have indicated that subjects with steatotic liver and NAFLD were more likely to have impaired glucose regulation and to develop T2DM [11,12]. Similar to NAFLD, MAFLD is considered an important risk factor for T2DM development, with authors reporting a more than twofold increased risk of T2DM in individuals with diagnosed MAFLD compared to those without MAFLD [13]. Although in this study, the incidence of T2DM in subjects without MAFLD has not been evaluated, the authors of the current investigation previously reported a 6.4% T2DM cumulative incidence in 7.5 years follow-up for the Spanish adult overall population of the nation-wide cohort Di@bet.es study; therefore, considering that the present study with a subsample from the same Di@bet.es cohort study is focused only on those participants at risk of T2DM with MAFLD, our results of 13.7% of T2DM cumulative incidence after 7.5 years follow-up would suggest a substantial increment in the new cases of T2DM within this subpopulation when compared with individuals without MAFLD. This result would be in line with the results of those previous investigations reporting an increased risk of T2DM development associated with the presence of steatotic liver, NAFLD, or MAFLD [11,12,13].

Different hypotheses related to hepatic insulin resistance have been suggested to explain this association [4,38]. Elevated levels of diacylglycerol or ceramides might lead to an abnormal increase in liver insulin resistance [39]; high concentrations of circulating transaminases have also been significantly linked to a future increased risk of T2DM [11], and high glucose levels might contribute to promoting insulin resistance in hepatocytes by stimulation of the glucose transporter-4 (GLUT4) expression and by inhibiting phosphatidylinositol 3-kinase/protein kinase B (PI3K/AKT) and AMP-activated protein kinase (AMPK) pathway in hepatic cells [40]; nevertheless, the specific molecular mechanisms underlying this association are not completely understood.

In our study, adherence to MD was inversely associated with baseline insulin resistance in the complete population with MAFLD and the group of individuals without weight gain at follow-up. This association is in keeping with a previous investigation in which factors cross-sectionally associated with insulin resistance were explored in 334 non-diabetic patients with NAFLD and where the MD score appeared inversely associated with insulin resistance independently of different covariables [41]. Also consistent with our result, different randomized interventional studies have demonstrated the benefits of MD, compared to other nutritional interventions on insulin-resistant improvement in obese and overweight patients and in patients with metabolic syndrome and NAFLD [9,24]. Among the different mechanisms suggested to explain the inverse association between MD intake and insulin resistance, Park et al. pointed to abdominal obesity as a mediating factor [42], suggesting that changes in waist circumference, more than in BMI, may play an important role in the pathways through which MD modulates insulin resistance. In the present study, baseline waist circumference and BMI were not different regarding MD adherence in the group of individuals who presented weight increment at follow-up, which might be related to the lack of association also observed between MD adherence and baseline insulin resistance categories in this group of individuals.

The positive effects of MD in the prevention of several co-morbidities related to fatty liver, such as insulin resistance, visceral obesity, dyslipidemia, and chronic inflammation, have been widely investigated [9,24]; however, to our knowledge, the relationship between adherence to MD and T2DM onset in subjects with MAFLD has not been evaluated so far, and only one study has investigated it in subjects with NAFLD [25]. In that study, Kouvari et al. [25] assessed the association of MD with NAFLD and their interaction in predicting ten-year T2DM onset in 1485 adult Greek subjects. They found for the total sample and stratified by sex that, while participants with NAFLD and an MD score below the median had around a three-times higher risk of developing T2DM than those without liver steatosis, those who adhered to MD were protected from an altered glycemic profile. In line with Kouvari et al.’s [25] study, our results have shown an inverse association between the adherence to MD and the risk of T2DM development in the Spanish adult population with MAFLD independently of the model adjustment; however, when the population was stratified by sex, this association was found significant only in men. The effect sizes of the high adherence to MD among sex, estimated by the ORs for the logistic regression models, are slightly lower in the group of women, which led us to believe that the sample size in this group of individuals could explain the lack of significant association; however, there are also potentially sexually dimorphic elements in fatty liver pathology, especially among people with obesity, such as the ketone body production from fatty acids partition, the synthesis of the very low-density lipoprotein cholesterol, fatty acid oxidation, or deposition of triglycerides as lipid droplets [43] that may also be related to this attenuated effect of MD in the group of women of our study. The biological mechanisms underlying the observed protecting role of MD might be the same as those involved in other metabolic improvements associated with MD intake in subjects with MAFLD or NAFLD, especially those related to the amelioration of the insulin resistance status, the main pathophysiologic condition linking fatty liver and T2DM [9]. The increment in the antioxidant capacity by following MD [44] may protect against oxidative stress, a crucial biological procedure in b-cell dysfunction and insulin resistance [45]. Additionally, MD patterns include different dietary nutrients and bioactive substances with anti-inflammatory and antimicrobial properties that have been associated with an improvement in insulin sensitivity and glucose metabolism [46]. Moreover, MD has a proven beneficial effect not only in long-term weight reduction [19] but also in fat-liver content reduction even without weight loss [21,23], which could lead to an increase in hepatic insulin clearance and insulin sensitivity, as well as the reduction in total body insulin resistance. All of these mechanisms potentially triggered by MD intake might act as a whole, alleviating the risk of T2DM.

It has been shown that MD had a beneficial role in the metabolic profile regardless of body weight loss [21,23]; however, in our study, although no significant interactions have been found between the adequacy of the MD and the weight gain, the protective role of the high adherence to MD on the risk of T2DM appeared evident in the group of subjects without weight gain at follow-up, while in the group of individuals with weight increment at follow-up, the inverse association between MD and the risk of T2DM did not rise significantly. A limited statistical power due to smaller sample size in the group of subjects who gained weight at follow-up could be related to this lack of significant association or interaction; however, considering the results showing that a 1 kg increase in body weight is associated with more than a 7% increase in the risk of type 2 diabetes [47], the possibility of a mitigated protective effect of MD on the T2DM development in the group of individuals who gained weight at follow-up by the opposite effect due to the weight increment cannot be ruled out.

The current study presents some limitations. Liver steatosis was assessed through the FLI score instead of a liver biopsy. This is a secondary analysis from the original diabetes study (starting in 2008) in which measurements for fat liver by ultrasound or other direct methods were not planned in its original design, so direct information for fatty liver is not available in this study. However, FLI is a non-invasive and well-established method for the diagnosis of liver steatosis, validated against ultrasound [29,30], that has also been recently validated as an MAFLD marker [31]. Results from Han AL’s study [31] verified the accuracy of the index for predicting MAFLD using abdominal computed tomography, and the author proposed FLI as a simple and cost-effective tool for clinical MAFLD screening. Nevertheless, any misclassification due to the use of this marker instead of liver biopsies or MRI would be random misclassification, which could have only resulted in a reduction in effect estimates. The inclusion of weight gain in the analysis would have the potential of being a source of a collider bias due to the possible simultaneous effect of the Mediterranean diet and the use of glucose-lowering drugs (diagnostic criteria for incident type 2 diabetes) on the weight increment at follow-up; however, in our study, only 39 individuals were under medication for diabetes control during follow-up a consequently diagnosed as incident diabetes for this reason, with no association between weight gain, continuous or dichotomous, and taking these medications thus, the possibility of collider bias, in this case, would be considered negligible. The sample size might be limited to detecting significant associations in the group of women and subjects with weight increment at follow-up when the overall population is split by sex or weight gain; nevertheless, OR for the association between high adherence to MD and T2DM incidence among sex and weight gain categories were similar and with the same direction, which led us to believe that the lack of significant associations in these subpopulations might be likely due to restricted sample size; to avoid possible interferences related to sex or weight gain, we included both confounding variables in the multivariable models as appropriate. Although participation in the follow-up was 66% and the possible participation bias in the whole study was minimal [27], the influence of some potential confounding factors cannot be completely excluded. Although differences resulting from interventions carried out in the participants’ home country have not been explicitly assessed, less variability is anticipated in data and sample collection because all procedures were carried out by the same qualified nurses at baseline and during follow-up. Although any physical or dietary recommendations were given to the participants by the researcher, it is not possible to know whether they received any later advice that might affect their body weight during the follow-up period; however, the likelihood of participants screened to subsequently change dietary/exercises habits in a way that could alter our final outcomes would be very low; even in those instances, only an attenuation of our effect estimates would be expected. These limitations are compensated by several strengths. This is the first established large prospective cohort that jointly evaluated any effect of adherence to MD and MAFLD on the long-term incidence of T2DM. Data for this investigation were obtained from a sizable nation-wide cohort, with a substantial duration of follow-up and a considerable number of events. OGTT was used to screen for the T2DM diagnosis in most of the participants, and HbA1c was also taken into account in the follow-up, which ensured the capture of most of the incident T2DM. A wide standardized test was used to evaluate the physical activity of the participants, and additionally, detailed dietary consumption data and information on MD adherence by the adapted 14-point MedDiet adherence screener (MEDAS) questionnaire [34] were obtained.

## 5. Conclusions

In conclusion, this prospective observational study of a Spanish representative cohort points to certain directions and raises hypotheses regarding the protecting role of the high adherence to MD on T2DM risk in subjects with MAFLD, with these beneficial effects more evident in men with the absence of weight increment. Altogether, our results might have implications in terms of clinical recommendations by supporting the nutritional recommendations about the utility of MD as the dietary pattern of choice in MAFLD patients [18] and also as preventive therapy for the development of T2DM; however, large, rigorously designed randomized clinical trials focused on the basis of our findings are needed in order to fully confirm the efficacy of MD, especially in non-Mediterranean populations and in comparison with other healthy dietary patterns.

## Figures and Tables

**Table 1:** Baseline general clinical characteristics according to level of adherence to Mediterranean Diet in overall population and population stratified by weight gain.

	Overall Study Population (n = 714)			
	n = 714	High Adherence (n = 265)	Low Adherence (n = 449)	*p*-Value *
Age in years (%)				<0.001
18–30	5.7	2.3	7.8	
31–45	25.2	20.0	29.0	
46–60	36.7	37.5	36.0	
61–75	27.5	33.6	24.0	
>75	4.9	6.4	4.0	
Sex (n (% men))	398 (55.7)	153 (57.7)	245 (54.6)	0.41
BMI (kg/m^2^)	32.18 ± 4.20	31.44 ± 3.88	32.61 ± 4.32	<0.01
Weight (kg)	86.80 ± 13.41	84.40 ± 12.23	88.22 ± 13.88	<0.01
Waist circumference (cm)	105.19 ± 9.42	104.27 ± 9.32	105.73 ± 9.44	<0.01
Fasting glucose (mg/dL)	97.44 ± 10.76	97.65 ± 10.53	97.31 ± 10.90	0.77
Fasting insulin (mU/dl)	11.75 ± 6.09	11.19 ± 6.34	12.07 ± 5.92	0.30
HOMA index	2.87 ± 1.67	2.74 ± 1.80	2.94 ± 1.58	0.22
Total cholesterol (mg/dL)	207.64 ± 37.20	210.88 ± 38.36	205.73 ± 36.41	0.16
HDL cholesterol (mg/dL)	47.72 ± 11.08	48.42 ± 11.54	47.30 ± 10.80	0.34
LDL cholesterol (mg/dL)	114.83 ± 27.66	115.92 ± 28.53	114.18 ± 27.15	0.58
Triacylglycerides (mg/dL)	162.99 ± 122.32	164.56 ± 139.19	160.06 ± 111.32	0.60
GOT (U/L)	20.15 ± 9.04	20.38 ± 8.31	20.02 ± 9.46	0.37
GPT (U/L)	18.58 ± 13.60	18.50 ± 12.59	18.64 ± 14.18	0.41
GGT (U/L)	44.40 ± 44.34	44.40 ± 39.17	44.39 ± 47.17	0.72
hsCRP (mg/L)	4.07 ± 4.78	4.23 ± 5.62	3.97 ± 4.21	0.67
Systolic blood pressure (mmHg)	137.80 ± 16.76	139.14 ± 16.57	137.01 ± 16.83	0.72
Diastolic blood pressure (mmHg)	81.44 ± 9.80	81.71 ± 9.50	81.27 ± 9.97	0.74
Metabolic syndrome (%)	65.4	66.8	64.6	0.55
Hypertension (%)	77.9	78.5	77.5	0.76
Obesity (%)	66.4	61.1	70.0	<0.01
Abdominal obesity (%)	79.1	78.1	79.7	0.60
Prediabetes (%)	50	49.4	45.7	0.39
Insulin resistance (%)	50.1	44.3	53.3	0.02
Dyslipidemia (%)	81.1	80.8	81.3	0.86
Steatogenic medication (%)	11.3	12.5	10.7	0.47
SF-IPAQ score (%)				0.04
Low	50.0	44.7	53.1	
Moderate	32.0	37.5	28.8	
High	18.0	17.8	18.1	
Smoking (%current smoker)	23.5	20.0	25.6	0.09
Alcohol consumption (%)				0.06
Never	22.5	19.6	24.3	
Low	8.1	6.8	8.9	
Moderate	50.3	49.8	50.6	
High	19.1	23.8	16.2	

Data presented as mean ± standard deviation or proportions. * Differences according to the MD adherence measured by univariant generalized linear model adjusted by age, sex, and abdominal obesity or chi-square test. BMI: body mass index; HDL: high-density lipoprotein; LDL: low-density lipoprotein; HOMA: homeostasis model assessment; hsCRP: high-sensitivity C-reactive protein; SF-IPAQ score: Short Form of International Physical Activity Questionnaire Score.

**Table 2:** Baseline general clinical characteristics according to level of adherence to Mediterranean diet in population stratified by weight gain.

	Without Weight Increment (n = 377)			Weight Increment (n = 337)		
	High Adherence (n = 135)	Low Adherence (n = 242)	*p*-Value *	High Adherence (n = 130)	Low Adherence (n = 207)	*p*-Value *
Age in years (%)			<0.01			0.04
18–30	0.7	7.4		3.8	8.2	
31–45	14.1	25.6		25.4	31.9	
46–60	37.8	32.2		37.7	40.6	
61–75	40.0	29.3		27.7	16.9	
>75	7.4	5.4		5.4	2.4	
Sex (n (% men))	65 (48.1)	115 (47.5)	0.90	88 (67.7)	130 (62.8)	0.36
BMI (kg/m^2^)	31.16 ± 3.46	33.26 ± 4.52	<0.001	31.74 ± 4.26	31.86 ± 3.96	0.90
Weight (kg)	82.49 ± 10.77	88.76 ± 14.18	<0.001	86.38 ± 13.35	87.58 ± 13.53	0.68
Waist circumference (cm)	103.50 ± 8.76	106.74 ± 9.41	<0.001	105.08 ± 9.83	104.56 ± 9.34	0.94
Fasting glucose (mg/dL)	96.86 ± 10.72	98.05 ± 11.40	0.19	98.48 ± 10.31	96.44 ± 10.26	0.25
Fasting insulin (mU/dL)	10.82 ± 5.08	12.45 ± 6.04	0.08	11.57 ± 7.41	11.63 ± 5.76	0.75
HOMA index	2.60 ± 1.35	3.06 ± 1.67	0.09	2.88 ± 2.16	2.79 ± 1.47	0.96
Total cholesterol (mg/dL)	213.71 ± 41.30	204.12 ± 35.78	0.06	207.94 ± 34.98	207.63 ± 37.12	0.93
HDL cholesterol (mg/dL)	49.53 ± 11.87	47.24 ± 9.96	0.17	47.26 ± 11.12	47.38 ± 11.73	0.92
LDL cholesterol (mg/dL)	117.09 ± 30.70	113.86 ± 27.47	0.49	114.67 ± 26.08	114.57 ± 26.85	0.92
Triacylglycerides (mg/dL)	156.07 ± 59.46	154.79 ± 74.04	0.39	173.38 ± 180.27	170.56 ± 140.85	0.97
GOT (U/L)	21.42 ± 9.42	19.62 ± 8.96	0.01	19.28 ± 6.82	20.50 ± 10.01	0.31
GPT (U/L)	18.85 ± 11.93	18.51 ± 16.07	0.07	18.12 ± 12.27	18.78 ± 11.67	0.60
GGT (U/L)	46.30 ± 41.81	40.80 ± 48.72	0.02	42.43 ± 36.28	48.59 ± 45.05	0.08
hsCRP (mg/L)	4.35 ± 5.59	3.93 ± 3.69	0.96	4.11 ± 5.67	4.01 ± 4.75	0.55
Systolic blood pressure (mmHg)	140.76 ± 16.34	138.42 ± 17.32	0.65	137.46 ± 16.71	135.36 ± 16.13	0.88
Diastolic blood pressure (mmHg)	82.72 ± 9.34	81.29 ± 10.01	0.25	80.66 ± 9.60	81.26 ± 9.95	0.33
Metabolic syndrome (%)	71.1	67.8	0.50	62.3	60.9	0.79
Hypertension (%)	84.4	78.1	0.13	72.3	76.8	0.35
Obesity (%)	60.7	75.2	<0.01	60.8	63.3	0.64
Abdominal obesity (%)	81.5	84.4	0.48	74.6	74.4	0.96
Prediabetes (%)	45.2	49.6	0.41	53.8	41.1	0.02
Insulin resistance (%)	43.9	55.0	0.04	44.6	51.5	0.22
Dyslipidemia (%)	85.2	79.3	0.16	76.2	83.6	0.09
Steatogenic medication (%)	13.3	12.0	0.70	11.5	9.2	0.48
SF-IPAQ score (%)			0.01			0.78
Low	42.2	54.8		47.3	51.2	
Moderate	42.2	27.8		32.6	30.0	
High	15.6	17.4		20.2	18.8	
Smoking (%current smoker)	14.8	21.5	0.11	25.4	30.4	0.31
Alcohol consumption (%)			0.06			0.05
Never	20.1	26.0		19.2	22.2	
Low	8.1	8.7		5.4	9.2	
Moderate	48.1	52.1		51.5	48.8	
High	23.7	13.2		23.8	19.8	

Data presented as mean ± standard deviation or proportions. * Differences according to the MD adherence measured by univariant generalized linear model adjusted by age, sex, and abdominal obesity or chi-square test. BMI: body mass index; HDL: high-density lipoprotein; LDL: low-density lipoprotein; HOMA: homeostasis model assessment; hsCRP: high-sensitivity C-reactive protein; SF-IPAQ score: Short Form of International Physical Activity Questionnaire Score.

**Table 3:** Incidence of T2DM according to the level of adherence to Mediterranean diet in the overall population and stratified by sex and weight gain at follow-up categories.

	New Cases (n (%))			
	All	High Adherence	Low Adherence	*p* *
Overall population (n = 714)	98 (13.7%)	28 (10.5%)	70 (15.6%)	0.06
Sex				
Men (n = 398)	50 (12.5%)	15 (9.8%)	35 (14.3%)	0.19
Women (n = 316)	48 (15.2%)	13 (11.6%)	35 (17.1%)	0.19
Weight gain at follow-up				
No weight increment (n = 377)	51 (13.5%)	11 (8.15%)	40 (16.5%)	0.02
Weight increment (n = 337)	47 (13.9%)	17 (13.1%)	30 (14.5%)	0.71

*: *p*-value for the differences according to adherence to the Mediterranean diet measured by chi-square test.

## Data Availability

The datasets presented in this article are not readily available because they are part of an ongoing study. Requests to access the datasets should be directed to the corresponding authors.

## Supplementary Materials

The following supporting information can be downloaded at https://www.mdpi.com/article/10.3390/nu16213788/s1, Table S1. Odd ratios and confidence interval for the association between adherence to Mediterranean diet (high vs. low) and incident T2DM in overall population and stratified by sex considering weight increment at follow-up as a continuous variable.
