# Supplementary material for: Protective Effect of High Adherence to Mediterranean Diet on the Risk of Incident Type-2 Diabetes in Subjects with MAFLD: The Di@bet.es Study
Source: Nutrients. 2024 Nov 4;16(21):3788. doi: 10.3390/nu16213788 (PMC11548257; doi:10.3390/nu16213788)
Supplement: Supplementary file 1 [file nutrients-16-03788-s001.zip › nutrients-3268870-supplementary.pdf]

**Supplementary Table S1.** Odd ratios and confidence interval for the association between adherence to Mediterranean diet (high vs low) and incident T2DM in overall population and stratified by sex considering weight increment at follow-up as a continuous variable.

| | **OR[95%CI]** | **p-value** |
|---|---|---|
| - Overall population | | |
| M1 | 0.51(0.31-0.85) | 0.01 |
| M2 | 0.52(0.31-0.87) | 0.01 |
| M3 | 0.48(0.28-0.81) | <0.01 |
| - Male | | |
| M1 | 0.46(0.23-0.93) | 0.03 |
| M2 | 0.48(0.24-0.96) | 0.04 |
| M3 | 0.44(0.21-0.91) | 0.03 |
| - Women | | |
| M1 | 0.59(0.28-1.24) | 0.16 |
| M2 | 0.61(0.29-1.30) | 0.20 |
| M3 | 0.56(0.26-1.23) | 0.15 |

M1: Logistic regression model for the risk of T2DM incidence adjusted by sex (except in the sex-based analysis), age (18-30, 31-45, 46-60, 61-75, >75), abdominal obesity, fasting serum glucose levels, family history of T2DM and weight gain (as continuous variable).

M2: M1 + insulin resistance index, hypertension, dyslipidemia and steatogenic medication.

M3: M2 + lifestyle variables (smoking habits, alcohol consumption and physical activity).
